# Familial Pancreatic Cancer: The Case for Prophylactic Pancreatectomy in Lieu of Serial Screening and Shared Decision Making

**DOI:** 10.1155/2014/737183

**Published:** 2014-11-20

**Authors:** Mazen E. Iskandar, Michael G. Wayne, Justin G. Steele, Avram M. Cooperman

**Affiliations:** ^1^Department of Surgery, Mount Sinai Beth Israel Medical Center, First Avenue at 16th Street, New York, NY 10003, USA; ^2^Department of Surgery, Mount Sinai Beth Israel Medical Center and The Pancreas and Biliary Center of New York, 37 Union Square West, New York, NY 10001, USA

## Abstract

At-risk family members with familial pancreatic cancer (FCaP) face uncertainty regarding the individual risk of developing pancreatic cancer (CaP) and whether to choose serial screening or prophylactic pancreatectomy to avoid CaP. We treated 2 at-risk siblings with a history of FCaP, congenital hepatic fibrosis (CHF), and jaundice secondary to a bile duct stricture. In one, a pancreaticoduodenal resection was done and in the second a total pancreatectomy. Malignancy was not present, but extensive pancreatic intraepithelial neoplasia (PanIn) 2 was present throughout both pancreata. The clinical course and literature review are presented along with the previously unreported association of CHF and CaP.

## 1. Case Report

Two siblings, a brother and a sister with a recent diagnosis of congenital hepatic fibrosis CHF and a strong family history of cancer, presented because of abdominal pain, jaundice, and abnormal liver function tests. Their family history was significant for fatal pancreatic cancer in their father and paternal uncle, colon cancer in their grandfather, and multiple myeloma in their mother. The 48-year-old sister was diagnosed first with CHF based on a liver biopsy after having abdominal pain and elevated liver enzymes. Within 3 months, she developed cholangitis. A computed tomography (CT) scan showed a dilated common bile duct (CBD) without stones, but no pancreatic mass. No masses or stones were also seen on endoscopic ultrasound (EUS). An endoscopic retrograde cholangiopancreatography (ERCP) and sphincterotomy and stenting of the CBD were then performed with a negative cytological examination of the bile duct brushings. Two additional attacks of cholangitis developed within 2 months. Because of recurrent cholangitis, the strong family history, and the uncertainty that screening would prevent her from developing CaP, she underwent a Whipple resection on July 17, 2012. The resected specimen showed no cancer but chronic pancreatitis with extensive PanIN 2. While the pancreas was being monitored, the hepatic fibrosis progressed and she has recently undergone a liver transplant and is doing well.

Her brother, 50 years old, experienced episodic right upper quadrant pain and elevated liver enzymes with a dilated bile duct, ascites on CT scan, and no gallstones. An MRCP demonstrated a distal CBD stricture and no pancreatic mass. An infiltrative process involved his liver, which on biopsy showed a variant of CHF, fibropolycystic disease. EUS/ERCP revealed a common bile duct CBD stricture and a benign ampullary polyp. The stricture was stented, the polyp is snared, and duct brushings were negative for malignant cells. In view of uncertain risk and his sister's pathology, he opted for a total pancreatectomy preferring the risks and close monitoring of endocrine and exocrine insufficiency to the risk of developing CaP. He underwent a total pancreatectomy and splenectomy on September 11, 2012, and chronic pancreatitis with multifocal PanIN 2, but no cancer was found. He is well on follow-up and on an insulin pump adjusting to life without a pancreas.

## 2. Discussion

Cancer of the pancreas remains a lethal disease with fewer than 5% of patients who actually survive 5 or more years [1]. The survival statistics remain abysmal despite the plethora of endoscopic and body imaging now available. Familial pancreatic cancer (FCaP) implies that 2 or more first degree relatives had CaP while sporadic CaP implies just one without satisfying the criteria of other cancer syndromes [2]. FCaP accounts for 3–10% of all cases of CaP [3]. In FCaP, the risk of CaP increases with the number of relatives affected. With 1, 2, and 3 affected family members, the risk is 4.6, 6.4, and 32 times for at-risk individuals [4]. Others at risk for CaP include those with polyposis syndromes such as Peutz-Jeghers (relative risk RR 132), hereditary pancreatitis (RR 53–87), familial atypical mole melanoma FAMM (RR 13–22), hereditary breast and ovarian cancer HBOC (RR 4–13), and hereditary nonpolyposis colon cancer, HNPCC (RR 4.5) [3]. Even when abnormal genes are expressed, the incidence of CaP is unknown.

The concern for every high risk individual (HRI) is how and how often to be screened and whether screening is capable of detecting an “early” malignancy and when to consider preventive pancreatectomy. Screening to detect CaP is expensive, insensitive, and dependent on a detectable mass. A century of seeking early, curable CaP has been fruitless. Even small malignancies seen on body imaging have a high incidence of metastases. Added to the expense of imaging tests and its low yield is the unknown risk of the development of an interval cancer between screening studies. This has been well studied by Tanaka et al. who evaluated over 27,000 patients referred for abdominal symptoms [5]. The screening study included a thorough abdominal sonogram with the evaluation of the pancreas and pancreatic duct. They then followed up 1058 patients with sonographic pancreatic abnormalities excluding those with known malignancies, for a mean of 75.5 months. Twelve patients developed CaP of whom 6 died. Screening showed 2 findings in the CaP patients: a dilated main pancreatic duct (MPD) of 2 or more mm and a cyst 5 mm or greater. However, 52% and 23% of the 1058 patients had a dilated MPD or a cyst >5 mm, making the positive yield of these lesions very small.

The value of screening should be directed to identification of premalignant lesions rather than early stage CaP. The “International Cancer of the Pancreas Screening Consortium” CAPS attempted to set criteria that are worth reviewing. The panel reviewed 12 published articles that studied the diagnostic yield and risk of developing cancer in 1040 combined HRIs mostly from the John's Hopkins and German registries [6]. After screening, 70/1040 patients developed masses and underwent resection, out of which 20 were pancreatic cancer. The outcome of these 20 cancers found on screening was disappointing if not dismal. Despite the goal of “early detection,” 9 patients died within less than 2 years, 3 others have recurrence or metastasis, and 2 are alive in short term follow-up [7–12]. There was no follow-up information on the remaining 6. There was a consensus that the best initial screening modality would be an EUS or a magnetic resonance cholangiopancreatogram (MRCP) with a diagnostic yield of around 43% [13]. However, no consensus was reached on proper modality or interval of follow-up or when surgery would be recommended [6].

The development of an interval cancer between screens indicates an aggressive tumor, assuming the lesion was not overlooked on a prior scan. Interval breast cancers, for example, have worse prognosis than those detected at initial screening [14]. The increased use of CT scan has increased detection of asymptomatic periampullary and pancreatic lesions which account for 6% of pancreaticoduodenectomies [15]. Incidental asymptomatic CaP may have a slightly longer survival than symptomatic lesions (30 versus 21 months in resected patients), but the survival advantage may be due to lead time bias [15].

The solution might lie in identifying accurate biologic markers. Serial evaluations of tumor markers such as Ca 19-9 indicate response to therapy but are otherwise nonspecific [16]. The study of pancreatic secretions for mitochondrial DNA and promoter DNA methylation mutations is not readily available and its value and accuracy require further study [17, 18]. Until specific precursor detection is available, total pancreatectomy is a strong alternative in HRIs.

The association between CHF and FCaP is unusual. CHF is an autosomal recessive disease characterized by fibrotic and abnormally proliferating biliary ducts leading to portal hypertension. Although CHF is mostly asymptomatic, some patients present with right upper quadrant pain or cholangitis. The most lethal complication of CHF is cholangiocarcinoma, but no association with pancreatic adenocarcinoma has been reported [19]. Jaundice in our 2 patients was due to a benign bile duct stricture secondary to pancreatitis associated with diffuse PanIN lesions.

## 3. Conclusion

Two high risk siblings with CHF and FCaP underwent partial and total pancreatectomy because of a bile duct lesion and strong family history of CaP. Pathology confirmed the presence of premalignant PanIN lesions. Both patients feared fatal outcome if CaP was detected, a very valid concern. Until sensitive markers for precursor lesions are available, partial or total pancreatectomy, before CaP is clinically evident, remains a reasonable consideration in HRIs and should be included in the decision making process when counseling patients.
